# Bendiocarb, a potential alternative against pyrethroid resistant *Anopheles gambiae *in Benin, West Africa

**DOI:** 10.1186/1475-2875-9-204

**Published:** 2010-07-14

**Authors:** Martin C Akogbéto, Gil Germain Padonou, Dina Gbénou, Seth Irish, Anges Yadouleton

**Affiliations:** 1 Centre de Recherche Entomologique de Cotonou (CREC), Cotonou, Bénin 06 BP: 2604; 2 London School of Hygiene & Tropical Medicine, London, WC1E 7HT, UK; 3 World Health Organization Office, Cotonou, Bénin

## Abstract

**Background:**

*Anopheles gambiae*, the main malaria vector in Benin has developed high level of resistance to pyrethroid insecticides, which is a serious concern to the future use of long-lasting insecticidal nets (LLIN) and indoor residual spraying (IRS). In this context, one of the pathways available for malaria vector control would be to investigate alternative classes of insecticides with different mode of action than that of pyrethroids. The goal of this study was to evaluate under field conditions the efficacy of a carbamate (bendiocarb) and an organophosphate (fenitrothion) against pyrethroid-resistant *An. gambiae s.s*.

**Methods:**

Wild populations and females from laboratory colonies of five days old *An. gambiae *were bio-assayed during this study. Two pyrethroids (deltamethrin and alphacypermethrin), an organophosphate (fenitrothion), a carbamate (bendiocarb) and a mixture of an organophosphate (chlorpyriphos + a pyrethroid deltamethrin) were compared in experimental huts as IRS treatments. Insecticides were applied in the huts using a hand-operated compression sprayer. The deterrency, exophily, blood feeding rate and mortality induced by these insecticides against *An. gambiae *were compared to the untreated control huts.

**Results:**

Deltamethrin, alphacypermethrin and bendiocarb treatment significantly reduced mosquito entry into the huts (p < 0.05) compared to untreated huts. Blood feeding rates in huts treated with fenitrothion and the mixture chlorpyriphos/deltamethrin were reduced from 10.95% respectively to 3.7% and 4.47% three months after treatment and from 10.20% to 4.4% and 2.04% four months after treatment. Exophily rates in huts with deltamethrin, alphacypermethrin and the mixture chlorpyriphos/deltamethrin were significantly higher than in the huts with fenitrothion. Deltamethrin and alphacypermethrin had the lowest mortality rate while fenitrothion killed 100% of *An. gambiae *(in the first month) and 77.8% (in the fourth month). Bendiocarb and the mixture chlorpyriphos/deltamethrin mortality rates ranged from 97.9 to 100% the first month and 77.7-88% the third month respectively.

**Conclusion:**

After four months, fenitrothion, bendiocarb and the mixture chlorpyriphos/deltamethrin performed effectively against pyrethroid-resistant *Anopheles*. These results showed that bendiocarb could be recommended as an effective insecticide for use in IRS operations in Benin, particularly as the mixture chlorpyriphos/deltamethrin does not have WHOPES authorization and complaints were mentioned by the sleepers about the safety and smell of fenitrothion.

## Background

In Benin, malaria is one of the most frequently recorded diseases in health centres. The incidence for both uncomplicated and complicated cases in 2006 was 139 per 1,000 inhabitants [1]. During the same year, malaria was the primary cause of mortality and morbidity in health centres in the departments of Ouémé and Plateau [1]. Over the past few years, the National Malaria Control Programme (NMCP) has implemented control interventions to reduce the contact between malaria vectors and human hosts. The major control strategies applied at national level were the scaling up of long-lasting insecticidal nets (LLINs) throughout the country and indoor residual spraying (IRS) in one of the cities of Benin (Cotonou). Despite these tremendous efforts made by the NMCP, the results obtained were less encouraging than expected. Resistance was suspected to be one of the reasons of the failure of malaria vector control programmes in Benin. In 1963, the World Health Organization reported that 32 species of *Anophelinae *were resistant in Africa to DDT and dieldrin [2]. After, Elissa reported the first case of pyrethroid resistance in *An. gambiae *in Côte d'Ivoire [3]. In other regions of Africa, numerous cases were documented in Kenya [4], Burkina Faso [3,5], South Africa [6], Côte d'Ivoire [3], Mali [7] and Cameroon[8]. In Benin, the knockdown gene implicated in resistance to DDT and pyrethroids was detected at high frequency (*kdr *> 0.9), especially in the urban areas of Cotonou [9-11]. A recent study by N'guessan *et al *in an experimental hut study showed a reduced efficacy of lambdacyhalothrin-treated nets against *An. gambiae *in Ladji, an outskirt area of Cotonou [12].

Despite these reports on pyrethroid resistance in Bénin, the National Malaria Control Programme decided to undertake, in 2008, a distribution of LLINs and to implement IRS in the department of Ouémé particularly in the districts of Sèmè-Kpodji, Dangbo, Missérété and Adjohoun. The increasing emergence of resistance leads to an urgent need to investigate alternatives to pyrethroid insecticides [13] and a continual monitoring of resistance before the implementation of any vector control programme. The widespread pyrethroid resistance is becoming a major problem faced by several National Malaria Control Programmes throughout Africa, particularly in Benin where failure of ITNs and IRS has been reported in experimental huts [12]. Experimental hut studies have shown that certain organophosphates and carbamates were particularly effective on wild populations of pyrethroid resistant vectors [14]. Field trials with the carbamates propoxur and bendiocarb for indoor residual spraying treatment have been very effective against pyrethroid resistant malaria vectors [15]. Over the past few years, there was an increasing interest in testing these insecticides for public health purposes as alternatives to pyrethroids. In the present study, we compare under semi field conditions in experimental huts the efficacy of the carbamate bendiocarb, the organophosphate fenitrothion and a mixture of chlorpyrifos(organophosphate) and deltamethrin (pyrethroid) for IRS treatment against pyrethroid resistant mosquitoes.

## Methods

### Study site

The study was carried out in experimental huts located in Akron at the outskirt of Porto Novo, [15], a swampy area used annually for vegetables cropping. There are many *An. gambiae *and other culicinae breeding sites around the vegetable plots. Porto Novo is situated at 6. 33 N, 2.37 E in the southern part of Benin, about 30 kilometres from the Atlantic Ocean. The composition of *An. gambiae s.l. *is 100% *An. gambiae s.s. *M form (Padonou, unpublished) the main malaria vector in this area. This vector is present all year-round and has developed a strong resistance to pyrethroid insecticides with a high frequency of *kdr *gene at 86.7% [16].

### Experimental huts

**Figure 1:**
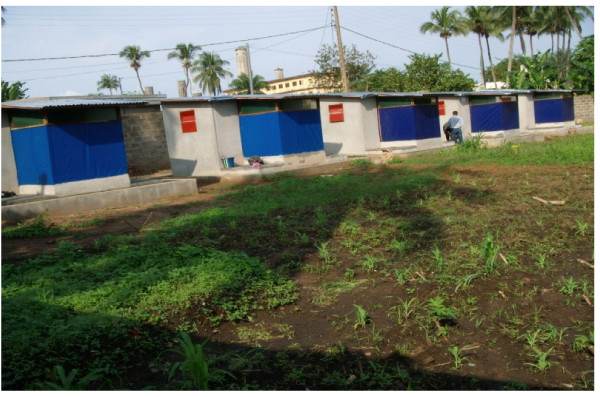
**Experimental huts, Akron station**. Courtesy *CREC (September 2007)*.

The study was conducted in experimental huts (Figure 1), which are designed for the standard WHO Phase II evaluation of insecticide-treated nets and IRS [17]. Experimental huts were originally used to study the behaviour of mosquitoes inside houses, but also used to evaluate the effect of IRS [18]. The experimental huts used were the West African type, of the Darriet model, that allows the entry of mosquitoes through the slits but not their exit. Each hut was 2.5 m long, 1.75 m wide, and had an interior ceiling 2 m high. The walls were made of concrete blocks covered with cement. The roof was made of corrugated iron. A tarpaulin was stretched under the roof to reduce heat in the hut and facilitate the capture of mosquitoes.

A 10 cm wide moat filled with water surrounded each hut to prevent the entry of scavengers such as ants and spiders. Six identical huts were built at the station. Five huts were treated with insecticides using a backpack sprayer and the sixth was left untreated as a control. The absorption of the walls was 112 ml of insecticide per m^2 ^and that of the ceiling (polyethylene), the entry slits, and the door (painted metal) was in total 53.13 ml/m^2^. The area of the walls to treat was 15 m^2 ^and that of the roof, the doors, and the entry slits was 5.1 m^2^. To treat the walls of the huts, 1.7 L of water was used. For the rest of the huts, 270.8 ml of water was used. Using these measures, the five huts were treated according to WHO recommendations [19];

Hut 1: Bendiocarb (800 g/kg) at 200 mg/m^2^;

Hut 2: Deltamethrin (250 g/kg) at 25 mg/m^2^;

Hut 3: Alphacypermethrin (50 g/kg at 30 mg/m^2^

Hut 4: Fenitrothion (400 g/kg) at 2 g/m^2^

Hut 5: Mixture of chlorpyriphos 250 g/L + deltamethrin 12 g/L at 560 mg/m^2 ^and 25 mg/m^2^, respectively

Throughout the study, sleepers slept under untreated bed nets.

### Biological materials

The behaviour of mosquitoes in the presence of insecticidal treatments was analysed on two samples of *An. gambiae*: The wild populations of Akron area attracted to sleepers inside experimental huts and wild *An. gambiae *emerged from field collected larvae which were released into the experimental huts. This last sample of mosquitoes was released during the period where there was insignificant density of mosquitoes entering the huts. The released *An. gambiae *were collected as larvae from the study site, and reared at the same place, so that the tested mosquitoes are not different from the wild population entering the huts. On average 20-27 not-blood-fed females of *An. gambiae *were released three times a month at 20:00 hours for a total number of 60-80 *An. gambiae *per hut.

### Sleepers and mosquito collection

Before the beginning of the evaluation, a blank collection of mosquitoes was carried out during two weeks in the experimental huts to compare the natural attractiveness between huts. Sleepers spent the same number of nights in each hut. The collections were done by six volunteer adult men, recruited by the CREC from the study area. The collectors were rotated between the huts, sleeping under a mosquito net from 21:00 hours to 06:00 hours. At 06:00 hours, mosquitoes were collected in the hut, using a mouth aspirator in the veranda and the hut room. By 08:00 in the morning, collection in huts was completed. All mosquitoes were put in netted plastic cups and transferred to the laboratory for identification. Mosquitoes were identified into species using Coluzzi key [20] and recorded as dead or alive, fed or unfed. Live mosquitoes were held in plastic cups and delayed mortality was recorded after 24 h. The effects of each treatment were expressed relative to the control in terms of:

- Deterrence rate: percentage of reduction in the number of mosquitoes caught in treated hut relative to the number caught in the control hut;

- Exophily rate: percentage of mosquitoes that have escaped the hut and have taken refuge in the veranda trap divided by the total number of mosquitoes collected in the hut;

- Blood-feeding rate: percentage of blood fed mosquitoes collected divided by the total of mosquitoes collected in verandah and hut;

- Immediate mortality: percentage of dead mosquitoes collected in the morning compared to total mosquitoes collected in the hut;

- Overall mortality: general mortality: immediate mortality + delayed mortality recorded after 24 h.

### Collection of qualitative data

Over the course of the study, interviews were conducted to identify any side effects of insecticide treatments on the collectors. These interviews were conducted at the end of the first and fourth month of collection.

### Statistical analysis

The statistical analysis was conducted using SPSS (Version 16.0). The effect of treatments was evaluated using analysis of variance (ANOVA) to compare treatments to the control.

### Ethical approval

The present study received a formal approval from the Ministry of Health of Benin and the Entomological Research Centre of Cotonou (CREC). The consent of all volunteers was required before their participation in the study. Malaria prevention and curative treatments were provided to all sleepers in the huts who showed symptoms of the disease.

## Results

### Attractiveness of huts before treatment

**Figure 2:**
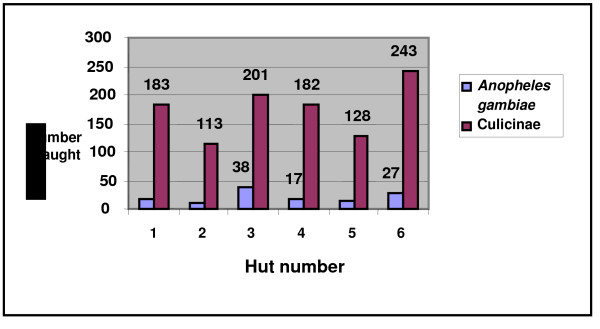
**Number of *An. gambiae *and culicinae mosquitoes collected in the experimental huts (September 5-17, 2007) before insecticidal treatment (Sleepers spent the same number of nights in each hut)**.

The homogeneity of attractiveness of the experimental huts was verified using ANOVA on the numbers of mosquitoes caught in each hut prior to treatment. This analysis showed (Figure 2) that the individual huts did not differ significantly in terms of the number of mosquitoes entering (p = 0.228 for *An. gambiae *and p = 0.257 for culicine mosquitoes).

### Results of collections after insecticide treatment

#### Deterrency and induced exophily

During the course of the first month after treatment, the huts treated with deltamethrin and alphacypermethrin had a significant deterrency for *An. gambiae *of 31.3% and 23.8%, respectively compared to the control hut (Table 1), and for culicinae of 46.2% and 38.4% (Table 2). The deterrency of the huts treated with bendiocarb, fenitrothion, and the chlorpyriphos-deltamethrin mixture were not significantly different from the control hut (p > 0.05). However, during the second month, the huts with deltamethrin and the mixture of chlorpyriphos and deltamethrin had shown a deterrency for *An. gambiae *of 24.8% and 30.7%, respectively (Table 2).

**Table 1:** Data of wild *Anopheles gambiae *collected 2 months post-treatment IRS (September to November 2007)

Treatments	Month	Number of females caught	Deterrency (%)	Exophily (%)		Blood feeding (%)		Immediate mortality (%)		Overall mortality (%)	
				Rate	Conf Lim	Rate	Conf Lim	Rate	Conf Lim	Rate	Conf Lim
**Control**	**1**	**80**	**-**	**12,5**^**a **^	**-**	**31,25**^**a **^	**-**	**0**^**a **^	**-**	**3,7**^**a **^	**-**
	2	101	-	19,80^a ^	-	29,70^a ^	-	0^a ^	-	2,97^a^	
**Bend**	**1**	**70**	**12,5**	**28,6**^**a**^	**[14,49-42,30]**	**20**^**a**^	**[12,13-31,85]**	**57,14**^**c**^	**[48,65-69,86]**	**92,85**^**c**^	**[85,76-99,65]**
	2	80	20,79	22,50^a^	[9,80-28,85]	12,50^b^	[3,05-19,05]	25^bc^	[10,93-41,82]	68,75^c^	[56,42-81,80]
**Delta**	**1**	**55**	**31,25**	**45,4**^**b**^	**[25,31-74,95]**	**18,18**^**a**^	**[6,28-33,91]**	**32,72**^**b**^	**[18,40-51,46]**	**72,72**^**b**^	**[58,79-84,06]**
	2	76	24,75	26,31^a^	[15,86-37,36]	23,68^a^	[7,04-50,63]	15,78^b^	[6,81-29,32]	31,57^b^	[22,81-40,33]
**A-Cyper**	**1**	**61**	**23,8**	**42,6**^**b **^	[24,62-61,36]	**19,67**^**a**^	**[6,65-33,45]**	**27,86**^**b**^	**[16,29-44,95]**	**77,04**^**b**^	**[59,85-95,52]**
	2	89	11,88	22,47^a^	[15,18-31,77]	13,48^a^	[6,88-21,50]	19,10^b^	[10,98-29,85]	39,32^b^	[34,33-44,32]
**Féni**	**1**	**85**	**0**	**24,7**^**a**^	**[13,16-39,70]**	**9,41**^**b**^	**[3,03-17,35]**	**91,76**^**d**^	**[84,30-96,87]**	**100**^**c**^	**[100-100]**
	2	95	5,94	22,10^a^	[14,20-28,93]	12,63^b^	[4,14-20,38]	40^cd^	[30,73-52,55]	73,68^c^	[64,41-82,95]
**Chlor- Delta**	**1**	**85**	**0**	**17,6**^**a**^	**[8,95-25,94]**	**10,58**^**b**^	**[3,01-22,59]**	**95,3**^**d**^	**[89,20-101,36]**	**100**^**c**^	**[100-100]**
	2	70	30,69	25,71^a^	[15,05-39,97]	11,42^b^	[4,02-17,35]	44,28^d^	[27,12-74,50]	78,57^c^	[62,06-95,00]

Bend: Bendiocarb; Delta: Deltamethrin; α-Cyper: Alpha-cypermethrin; Feni: Fenitrothion; Chlor- Delta: Chlorpyriphos + Deltamethrin.

Conf Lim: 95% confiance limits

For a same parameter of the table, values which carry different letters in exposant were significantly differents (p < 0. 05).

**Table 2:** Data of wild *Culex sp. and Mansonia sp. *one month after IRS treatment (September to October 2007).

Treatments	Number of females Catches	Deterrency (%)	Exophily (%)		Blood feeding (%)		Immediate mortality (%)		Overall mortality (%)	
			Rate	Conf Lim	Rate	Conf Lim	Rate	Conf Lim	Rate	Conf Lim
**Control**	975	-	30,76^a ^	-	19,48^a ^	-	0,30^a ^	-	2,56^a ^	-
**Bend**	550	43,58	27.27^a^	[23,00-32,53]	16,36^b^	[14,22-19,00]	19,09^c^	[15,84-23,02]	38,36^c^	[35,46-41,35]
**Delta**	525	46,15	33,33^a^	[28,67-38,33]	14,28^b^	[9,58-20,07]	8,5^b^	[7,07-10,10]	20,95^b^	[17,74-23,79]
**α-Cyper**	601	38,35	33,44^a^	[31,74-35,72]	16,63^b^	[9,24-24,02]	11,81^b^	[5,57-18,05]	24,29^b^	[14,57-33,72]
**Féni**	805	17,43	21,73^a^	[18,41-24,63]	14,90^b^	[13,91-15,77]	31,18^e^	[27,80-34,13]	53,41^d^	[50,14-56,37]
**Chlor- Delta**	850	12,82	27,05^a^	[25,10-29,01]	14,11^b^	[12,79-15,45]	23,64^d^	[22,26-24,86]	52,11^d^	[49,22-55,21]

Bend: Bendiocarb; Delta: Deltaméthrin; α-Cyper: Alpha-cyperméthrin; Féni: Fénitrothion; Chlor- Delta: Chlorpyriphos + Deltaméthrin.

Conf lim: 95% confiance limits

For a same parameter of the table, values which carry different letters in exposant were significantly differents *(p < 0, 05)*.

In addition to reduction of entry, the huts treated with deltamethrin and alphacypermethrin induced significant levels of exophily (p < 0.05) on wild *An. gambiae *(Table 1), as well as those released in the huts (Table 3). This was not the case with other treatments, which were not significantly different from the control (p > 0.05). However, a significant increase in exophily was noted with bendiocarb, fenitrothion and the chlorpyriphos-deltamethrin mixture (p < 0.05). In the third and fourth months, the exophily in huts treated with alphacypermethrin and the chlorpyriphos-deltamethrin mixture increased from 42.3 to 55.9% and from 35.8 to 57.1%, respectively.

**Table 3:** Data of *Anopheles gambiae *collected during 4 months after IRS treatment (September 2007 to January 2008)

Treatments	Month	Number of females caught	Exophily (%)		Blood feeding (%)		Immediate mortality (%)		Overall mortality (%)	
			Rate	Conf Lim	Rate	Conf Lim	Rate	Conf Lim	Rate	Conf Lim
	**1**	**62**	**16,12**^**a **^	**-**	**32,25**^**a **^	**-**	**0**^**a **^	**-**	**3,22**^**a **^	**-**
	2	73	27,39^a ^	-	13,69^a ^	-	0^a ^	-	2,73^a ^	-
**Control**	**3**	**73**	**6,84**^**a **^	**-**	**10,95**^**a **^	**-**	**0**^**a **^	**-**	**4,10**^**a **^	**-**
	4	49	42,85^a ^	-	10,20^a ^	-	0^a ^	-	6,12^a ^	-
	**1**	**60**	**25**^**b**^	**[21,92-28,16]**	**25**^**b**^	**[21,92-28,16]**	**50**^**e**^	**[43,86-56,31]**	**81,66**^**d**^	**[76,23-87,20]**
	2	48	33,33^b^	[28,28-38,31]	10,41^a^	[2,43-18,19]	72,91^d^	[67,44-78,50]	81,25^d^	[68,78-94,14]
**Bend**	**3**	**72**	**51,38**^**e**^	**[45,40-57,37]**	**8,33**^**a**^	**[8,33-8,33]**	**69,44**^**c**^	**[63,47-75,40]**	**77,77**^**c**^	**[71,79-83,76]**
	4	46	63,04^d^	[54,68-71,42]	4,34^a^	[-5,11-14,01]	10,86^b^	[1,82-19,85]	47,82^d^	[35,72-60,11]
**Delta**	**1**	**60**	**38,33 **^**c**^	**[29,55-47,26]**	**16,66**^**d**^	**[7,31-19,24]**	**25**^**b**^	**[21,92-28,16]**	**66,66**^**b**^	**[62,45-70,95]**
	2	52	71,15^d^	[68,80-73,47]	9,61^a^	[1,57-17,60]	7,69^b^	[-0,9385-16,4]	67,30^c^	[59,88-74,76]
	**3**	**63**	**23,80**^**b**^	**[23,81-23,81]**	**6,34**^**a**^	**[-0,48-13,17]**	**6,34**^**a**^	**[-0,48-13,17]**	**22,22**^**b**^	**[15,39-29,05]**
	4	32	46,87^ab^	[40,44-53,49]	9,37^a^	[8,09-10,70]	3,12^a^	[-10,00-16,07]	28,12^b^	[24,26-32,09]
	**1**	**60**	**35 **^**c**^	**[30,70-39,42]**	**18,33**^**c**^	**[10,71-26,02]**	**26,66**^**b**^	**[22,15-31,11]**	**78,33**^**c**^	**[71,68-81,75]**
	2	82	59,75^c^	[57,66-61,82]	12,19^a^	[7,60-16,73]	39,02^c^	[34,39-43,65]	60,97^b^	[56,34-65,60]
**α-Cyper**	**3**	**78**	**42,30**^**d**^	**[42,31-42,31]**	**5,12**^**a**^	**[-0,37-10,63]**	**3,84**^**a**^	**[3,85-3,85]**	**24,35**^**b**^	**[18,85-29,86]**
	4	34	55,88^bcd^	[38,81-73,31]	5,88^a^	[-6,97-19,10]	29,41^c^	[14,60-44,47]	35,29^c^	[31,01-39,70]
	**1**	**70**	**27,14**^**b**^	**[22,70-31,53]**	**24,28**^**b**^	**[18,65-29,90]**	**42,85**^**c**^	**[40,28-45,47]**	**85,71**^**d**^	**[80,54-90,96]**
	2	43	67,44^d^	[58,43-76,49]	11,62^a^	[1,95-21,23]	74,4^d^	[65,25-83,63]	83,72^d^	[75,62-91,99]
**Féni**	**3**	**54**	**22,22**^**b**^	**[19,18-25,34]**	**3,7**^**b**^	**[-4,31-11,73]**	**68,51**^**c**^	**[56,65-80,65]**	**90,74**^**d**^	**[76,47-105,37]**
	4	45	51,11^bc^	[41,55-60,66]	4,44^a^	[-5,11-14,01]	71,11^d^	[61,55-80,66]	77,77^f^	[65,99-85,11]
	**1**	**80**	**25**^**b**^	**[20,89-29,07]**	**26,25**^**b**^	**[24,84-27,68]**	**46,25**^**d**^	41,64-50,86]	**81,25**^**d**^	**[72,79-89,80]**
	2	68	58,82^c^	[53,40-64,26]	8,82^a^	[8,28-9,40]	70,58^d^	[66,09-75,15]	82,35^d^	[76,92-90,67]
**Chlor- Delta**	**3**	**67**	**35,82**^**c**^	**[33,56-38,09]**	**4,47**^**b**^	**[4,19-4,77]**	**61,19**^**b**^	**[55,50-66,89]**	**88,05**^**d**^	**[81,93-94,21]**
	4	49	57,14^cd^	[45,17-60,66]	2,04^b^	[-6,88-11,05]	28,57^c^	[20,57-36,53]	63,26^e^	[60,07-66,41]

Bend: Bendiocarb; Delta: Deltaméthrin; α-Cyper: Alpha-cyperméthrin; Féni: Fénitrothion; Chlor- Delta: Chlorpyriphos + Deltaméthrin.

Conf Lim: 95% confiance limits

For a same parameter of the table, values which carry different letters in exposant were significantly differents *(p < 0, 05)*.

#### Blood feeding

The blood feeding rates of wild Anopheles entering the huts treated with deltamethrin, alphacypermethrin, and bendiocarb were not significantly different from the control hut during the first month (p > 0.05) (Table 1). Contrary to what was observed with wild Anopheles, with the culicine and the released *An. gambiae*, the blood feeding rates in all five insecticide treatments were significantly less than that of the control (p < 0.05). In the second month, the blood feeding rates of mosquitoes in the Bendiocarb, fenitrothion and chlorpyriphos-deltamethrin mixture huts were significantly less than the control hut (p < 0.05). Over the third and fourth months, blood feeding in the chlorpyriphos-deltamethrin mixture and fenitrothion huts remained lower than that of the control (p < 0.05).

### Immediate mortality

During the first two months, the immediate mortality rates of mosquitoes in huts treated with deltamethrin and alphacypermethrin were significantly higher than the control (p < 0.05). During the second month (Table 3), the mortality of released *An. gambiae *in hut treated with alphacypermethrin (39.0%) was significantly higher than that treated with deltamethrin (p < 0.05). For bendiocarb, the immediate mortality during the first month was clearly higher than that of deltamethrin and alphacypermethrin, but less than that of those of fenitrothion and the mixture of chlorpyriphos and deltamethrin. During the second month, the immediate mortality in the chlropyriphos-deltamethrin hut (44.3% for wild *An. gambiae*, Table 1) remained unchanged. The released *An. gambiae*, in fenitrothion and the mixture chlorpyriphos-deltamethrin huts resulted in highest rates of immediate mortality, giving 74.4% and 70.6%, respectively. During the third and fourth months, there was a complete decline of the immediate mortality in huts with deltamethrin (6.4%) and alphacypermethrin (3.1%). However, the insecticidal effects of fenitrothion 68.6% and 71.1%, chlorpyriphos-deltamethrin 61.2% and 28.6%, alphacypermethrin 3.4% and 29.4%, and bendiocarb 69.5% and 10.9% remain continually high (Table 3).

#### Overall mortality

Over the first month, deltamethrin and alphacypermethrin showed good performance with high overall mortality of 72.1% and 77.0 on wild *An. gambiae *(Table 1). Fenitrothion and the chlorpyriphos-deltamethrin mixture had the highest mortality rates with 81.3% and 85.7%, respectively on wild *An. gambiae *(Table 3) and 52.1% and 53.4% on culicine (Table 2). A higher overall mortality rate was found in the hut treated with bendiocarb (92.8%), the chlorpyriphos-deltamethrin (100%), and fenitrothion (100%) on wild *An. gambiae*. During the second month, the effect of deltamethrin and alphacypermethrin was reduced to 31.6% and 39.3%, respectively. However, the residual effects of carbamates and organophosphates remained high until the third month with mortality rates of 77.8% for bendiocarb, 90.7% for fenitrothion and 88.1% for the mixture chlorpyriphos-deltamethrin. Over the fourth month, the overall mortality with deltamethrin treatment declined to 28.1% while fenitrothion had the highest rate at 77.7%, followed by the chlorpyriphos-deltamethrin mixture, bendiocarb, and alphacypermethrin.

#### Side effects of the treatment on sleepers

A regular follow up of side effects of the insecticide treatments on sleepers was conducted using a questionnaire. Certain complaints were registered during the first months when the sleepers spent the night in the huts treated with fenitrothion and the chlorpyriphos-deltamethrin mixture. The effects noted were irritating action to the eyes and nose.

But no negative effects were noted during the fourth month. However, the sleepers noticed that the treatments were reducing the biting nuisance of mosquitoes in the treated huts than in their own homes or the control hut. In response to the question "Would you like to continue the experiment?" all responded "yes."

## Discussion

The initial collections before the insecticide treatments in the huts revealed that the huts were not significantly different in their attractiveness. However, huts 2 and 5 collected the fewest mosquitoes. Hut 6 caught the most culicinae and hut 3 caught the most *An. gambiae*. These effects were probably due to the proximity of the huts to the larval sites.

The deterrency or reduction of entry rates for both *An. gambiae *and culicinae was the most evident factor observed in huts treated with the pyrethroids, alphacypermethrin and deltamethrin. As for fenitrothion and the chlorpyriphos-deltamethrin mixture, the reduction was very low and this may probably due to the fact that organophosphates have not repellent action compared with pyrethroids (deltamethrin and alphacypermethrin) [21]. However, the entry rate in the hut treated with bendiocarb was reduced compared to that of organophosphates. Natural exophily (in the control hut) of *An. gambiae *varied throughout the study. The lowest rate (6.8%) was observed in 3^rd ^month after treatment and the highest rate the following month (42.8%). It is very difficult to explain this variation in behaviour as both rates take place during the same period. These months correspond to the beginning of the dry season and do not seem to result from changes of behaviour in humans or mosquitoes.

The induced exophiliy (in the treated hut) of wild *An. gambiae *was the highest in huts where walls were treated with alphacypermethrin and deltamethrin as a result of the repellent effect pyrethroids, which was not observed with carbamates and organophosphates. In contrast, the induced exophily on released *An. gambiae *in huts treated with fenitrothion and the chlorpyriphos-deltamethrin mixture was relatively high. The strong exophily due to the carbamates and organophosphates on these specimens might be explained by the fact that the *An. gambiae *released in the huts had lost their vigour after being reared in the insectary. The effect of the chlorpyriphos-deltamethrin mixture could be explained by a synergy between the two products. Similar results were found when testing combinations of non-pyrethroid insecticides and a repellent (DEET), an organophosphate (chlorpyriphos methyl) and an oxadiazine (Indoxacarb) alone or in combination with pyrethroids on resistant mosquitoes [22-25].

The treatment of the huts with insecticide did not prevent a proportion of mosquitoes from taking a blood meal. This blood meal was facilitated by the fact that the mosquito nets used to protect sleepers were not treated. The fact that deltamethrin, alphacypermethrin, and bendiocarb did not significantly reduce blood feeding compared to the control might be explained by the significant immediate mortality in the other two insecticides during the first month, fenitrothion (91.7%) and the chlorpyriphos-deltamethrin mixture (95.3%) (p < 0.05). In these cases, mosquitoes entering the huts could also have been killed before being able to blood feed.

The mortality of culicinae was only 53.4% with fenitrothion in the first month; this can be explained by the resistance of culicinae, largely *Culex *and *Mansonia spp*. Resistance to organophosphates, carbamates, and pyrethroids has been reported in *Culex quinquefasciatus *in West Africa [9]. Moreover, in Ladji (south of Bénin) high frequencies of resistance to permethrin, DDT and carbosulfan were recorded in *Cx. quinquefasciatus *[26]. However, the *An. gambiae *were strongly affected by fenitrothion, the mixture of chlorpyriphos-deltamethrin, and bendiocarb. The difference between carbamates, organophosphates and pyrethroid insecticides can be explained by the current emergence and widespread resistance of *An*. *gambiae s.l*. to pyrethroids that we mentioned earlier [10]. Other than the complaints about fenitrothion and the chlorpyriphos-deltamethrin mixture that caused certain minor problems in the beginning of the first month, there was no other complaints recorded about the pyrethroids or bendiocarb afterwards. These experiences were noted and confirmed by the sleepers. It should be noted that chlorpyriphos is not recommended by the WHO for indoor residual spraying [20].

## Conclusion

After four months experiment of indoor residual spraying treatments in experimental huts, fenitrothion, chlorpyriphos-deltamethrin mixture, and bendiocarb were shown to be effective insecticides for controlling pyrethroid-resistant Anopheles. They showed to be effective alternatives to pyrethroids for indoor residual spraying. Bendiocarb decayed in less than four months, showing a short-life on cement walls, but still seems a promising insecticide to control resistant vectors. A micro-encapsulation formulation of bendiocarb will make it last longer in treated supports. Reports from Equatorial Guinea, Namibia, Mozambique, Mexico, and India showed good performance of bendiocarb as an indoor residual spraying treatment against mosquito vectors. The mixture of chlorpyriphos and deltamethrin is not yet registered with the WHO and cannot be imported for public health purposes. Fenitrothion was an effective product, but the side effects were not appreciated by the sleepers, however reports on fenitrothion indicated its better personal protection effects than Bendiocarb.

## Competing interests

The authors declare that they have no competing interests.

## Authors' contributions

**MCA **conceived and designed the study, supervised fields and laboratory procedures, data analysis and interpretation, revised the manuscript and gave final approval for the version to be published. **GGP **carried out field experiments, collected, analysed, interpreted data and wrote the first draft of the manuscript. **SI **helped with translation of the manuscript and contributed to the design of the study. **DG **and **AY **contributed to the design of the study and substantially helped in drafting the manuscript. All authors read and approved the final manuscript.
